# Is Aquatic Life Correlated with an Increased Hematocrit in Snakes?

**DOI:** 10.1371/journal.pone.0017077

**Published:** 2011-02-16

**Authors:** François Brischoux, Gabriel E. A. Gartner, Theodore Garland, Xavier Bonnet

**Affiliations:** 1 Centre d'Etudes Biologiques de Chizé – CNRS, 79360, Villiers en Bois, France; 2 Department of Biology, University of California Riverside, Riverside, California, United States of America; University of Queensland, Australia

## Abstract

**Background:**

Physiological adaptations that allow air-breathing vertebrates to remain underwater for long periods mainly involve modifications of the respiratory system, essentially through increased oxygen reserves. Physiological constraints on dive duration tend to be less critical for ectotherms than for endotherms because the former have lower mass-specific metabolic rates. Moreover, comparative studies between marine and terrestrial ectotherms have yet to show overall distinct physiological differences specifically associated with oxygen reserves.

**Methodology/Principal Findings:**

We used phylogenetically informed statistical models to test if habitat affects hematocrit (an indicator of blood oxygen stores) in snakes, a lineage that varies widely in habitat use. Our results indicate that both phylogenetic position (clade) and especially habitat are significant predictors of hematocrit. Our analysis also confirms the peculiar respiratory physiology of the marine *Acrochordus granulatus*.

**Conclusion/Significance:**

Contrary to previous findings, marine snakes have significantly–albeit slightly–elevated hematocrit, which should facilitate increased aerobic dive times. Longer dives could have consequences for foraging, mate searching, and predation risks. Alternatively, but not exclusively, increased Hct in marine species might also help to fuel other oxygen-demanding physiological adaptations, such as those involved in osmoregulation.

## Introduction

Evolutionary habitat transitions often involve substantial modifications in morphology, physiology, and behaviour, particularly if the new habitat imposes novel physical challenges. For example, transition to an aquatic life leads to selection on such attributes as the ability to move through water, to remain underwater for long periods, and to dive to considerable depths [1]. Features facilitating such tasks are different from those required in most terrestrial organisms. Accordingly, lineages of secondarily marine vertebrates often provide remarkable examples of specific adaptation to aquatic life [1]–[3].

Physiological adaptations that allow air-breathing vertebrates to remain underwater for long periods primarily involve modifications of the respiratory system, essentially through increased oxygen reserves (e.g. blood volume, hemoglobin concentration, myoglobin concentration [3]). Enlarged oxygen stores allow increased dive duration while delaying the shift toward anaerobic metabolism, which would force the animal to spend longer periods of time breathing and resting at the surface after dives to purge its physiological debt [3].

Previous research on physiological and morphological adaptations to aquatic life has mainly focused on endothermic vertebrates [4]. However, physiological constraints on diving should be less of a problem for ectotherms than endotherms, for at least three reasons [4]. First, ectotherms have relatively low mass-specific metabolic rates (compared to endotherms) and hence reduced oxygen needs [5]. Second, ectothermic vertebrates exhibit a marked flexibility in many if not most aspects of physiology, including those involved during extended breath-holding periods (e.g. body temperature, anoxia, acidosis, glycaemia [6], [7]). Finally, marine reptiles do not face the heat-conserving constraints on body shape imposed by endothermy, and they have high surface area-to-volume ratios that potentially allow for increased cutaneous oxygen uptake while submerged [8]–[11].

Most of the previous comparisons of terrestrial and diving, air-breathing, ectothermic “reptiles” have failed to find common physiological differences specifically associated with respiration and metabolism [9], [12]–[15]. In a few instances, however, studies have demonstrated taxon-specific physiological modifications that appear to represent evolutionary adaptations to aquatic life in ectotherms. For example, the highly aquatic Acrochordidae (especially the marine *Acrochordus granulatus*) have an exceptionally large oxygen store (combining both high blood volume and high blood oxygen carrying capacities [16]). In addition, leatherback turtles (*Dermochelys coriacea*) have high myoglobin concentration in their muscles [17]. Apart from these two extreme examples of marine reptiles displaying oxygen stores approaching levels found in mammals and birds, the failure to find common physiological adaptations in marine reptiles (as compared with studies of endotherms [3]) has lead to the notion that the primary adaptations for diving in ectotherms may be morphological (e.g. paddle-shaped limbs or tails) and behavioural, rather than physiological [9], [14], [15]. Nonetheless, previous empirical studies are far too limited to allow a clear understanding of how ectothermic vertebrates cope with the challenges associated with adoption of a secondarily aquatic lifestyle [4].

Snakes are well suited to identify possible evolutionary adaptations to aquatic life. First, almost every family has undergone independent transitions from terrestrial toward aquatic or marine habits, thus allowing examination of possible parallel or convergent physiological adaptations among lineages [13]. Second, snakes occupy a continuum of ecological situations between terrestrial and marine species within restricted phylogenetic lineages (i.e. the family [13]), thus allowing comparisons of closely related species along a gradient of habitat use. Third, because of a relative consistency in blood volume and hemoglobin-oxygen affinity between terrestrial and aquatic species [12]–[14], and the absence of significant amounts of myoglobin in snake muscle [14], [18], indexing blood oxygen stores by the simple measurement of hematocrit is reasonable [12]–[14], [18].

Here, we analyze hematocrit in relation to the use of aquatic environments in snakes by use of both phylogenetic and non-phylogenetic statistical models [19]–[21]. We also explicitly examine the relationship of hematocrit to both habitat use and phylogenetic position.

## Methods

Information on hematocrit (henceforth, Hct; measured as percent packed blood cell volume per unit volume of blood) was collected from the literature and from unpublished studies (67 species belonging to 7 families, Online Appendix S1). When multiple values of Hct were available for a given species, we used a mean value. Because Hct can vary with age in squamates [22]–[24], we retained Hct of adult snakes only in our analyses.

We assigned to each snake species a Habitat category based on published data, field guides, and personal experience (Online Appendix S1).

*Terrestrial*. Species without any particular affinity for water.

*Semi-aquatic*. Amphibious species associated with freshwater.

*Aquatic*. Fully aquatic species living in freshwater.

*Marine*. Species living almost exclusively at sea. Due to long foraging trips (i.e. weeks), which require developed diving capacities, amphibious Laticaudinae were included in this group.

Some populations of semi-aquatic species can be observed in xeric environments (e.g. *Notechis scutatus* [25]), but we emphasize that we selected the most typical Habitat for a given species, usually taking into account the location of the populations analysed for Hct in the literature.

**Figure 1:**
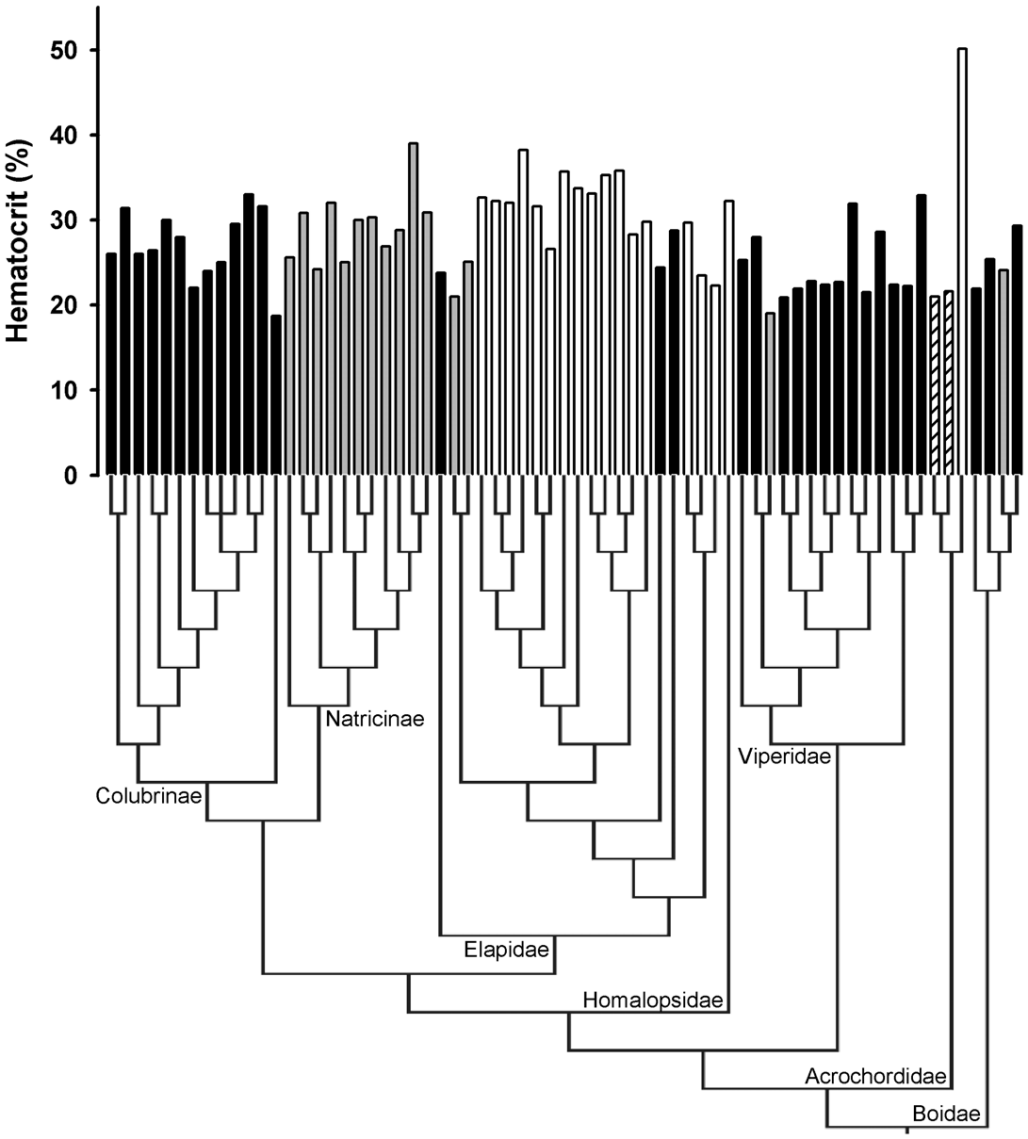
Phylogeny used for analyses with corresponding Hct for each species (see Online Appendix S1, S2 & S3 for details). Branch lengths represent the arbitrary method of Pagel, as used for statistical analyses. Black bars are for terrestrial species, light grey bars for semi-aquatic species, hatched bars for aquatic species, and white bars for marine species. Left-to-right order matches order of species in Online Appendix S1.

We constructed a composite estimate of phylogenetic relationships using hypotheses from previously published studies. We began with higher-level relationships connecting the major lineages of snakes, and nested lower-level relationships within this framework. We followed published methods for selecting among multiple trees for any given set of taxa ([21], [26] Figure 1 and Online Appendix S2 & S3 for details on the phylogeny construction).

We examined the effects of Clade and Habitat using conventional (non-phylogenetic) multiple regression (Ordinary-Least-Squares - OLS - regressions) with dummy variables that code for Clade and Habitat (ANCOVA with parallel slopes). We repeated the analysis using Phylogenetic Generalized Least Squares (PGLS) models and also with phylogenetic models that use a branch-length transformation based on the Ornstein-Uhlenbeck (OU) model of evolution for residual Hct variation (henceforth, RegOU [27]). In all cases, our alternate models increased in complexity (no independent variable, Clade or Habitat, Clade and Habitat: Table 1). We performed all multiple regressions in Matlab using Regressionv2.m [27]. The fit of alternate models was compared using their AICc (Akaike's Information Criterion -AIC - with a second order correction for small sample sizes, where smaller values indicate a better fit of the model to the data [28]); ln maximum likelihood ratio tests (LRT) were used to test for phylogenetic signal by comparison of the RegOU with the OLS models. Further details on the statistical procedures are available elsewhere [21], [27].

**Table 1:** Table of alternate regression models for predicting Hct in snakes.

	Conventional (OLS)						Phylogenetic (PGLS)						Phylogenetic with O-U model (RegOU)						
Model	Ln ML	AIC	AICc	MSE	SEE	r^2^	Ln ML	AIC	AICc	MSE	SEE	r^2^	Ln ML	AIC	AICc	MSE	SEE	REML d	r^2^
No independent variable	−209.29	422.59	422.78	30.71	5.54	0	−223.48	450.95	451.14	46.90	6.85	0	−208.24	422.47	422.85	29.77	5.46	0.124	0
Clade	−203.09	422.18	424.67	28.08	5.30	0.17	−223.15	462.29	464.77	51.09	7.15	0.01	−202.75	423.50	426.66	28.01	5.29	0.057	0.15
Habitat	−198.05	406.11	407.09	23.01	4.80	0.28	−207.65	425.30	426.29	30.64	5.53	0.38	−197.58	407.15	408.55	22.85	4.78	0.195	0.27
Clade + Habitat	**−183.20**	**388.39**	**393.19**	**16.32**	**4.04**	**0.54**	**−**205.36	432.72	437.52	31.62	5.62	0.41	**−**183.20	390.39	396.17	1.65	4.07	0.035	0.52

Based on AIC and AICc (smaller values indicate better-fitting and more parsimonious models), the OLS (Clade+Habitat) model (shown in boldface) is preferred. ML, MSE, SEE, and REML d stand for maximum likelihood, mean squared error, standard error of the estimate, and REML estimate of d (the OU transformation parameter), respectively. See text and [21], [27] for further explanations.

## Results

Based on AICc, the preferred model is OLS with both Clade and Habitat as independent variables (AICc = 393.19, Table 1). The model with the next-lowest AICc (396.17) is the RegOU with the same independent variables (Table 1). Based on the "rough rules of thumb" of Burnham and Anderson ([28] p. 70), the difference in AICc between these two models (∼3) would indicate that the RegOU model has "weaker support." All other models have AICc values that are at least 10 larger (Table 1), indicating "virtually no support".

Based on LRTs for models incorporating the same independent variables, the RegOU models never fit the data significantly better than the corresponding OLS models. However, the AICc of the RegOU model is always larger due to the extra parameter estimated in this model (i.e. the OU transformation parameter). Thus, we found no statistically significant phylogenetic signal in the residuals of our dependent variable after accounting for variation related to the Habitat and Clade independent variables [27], [29].

**Figure 2:**
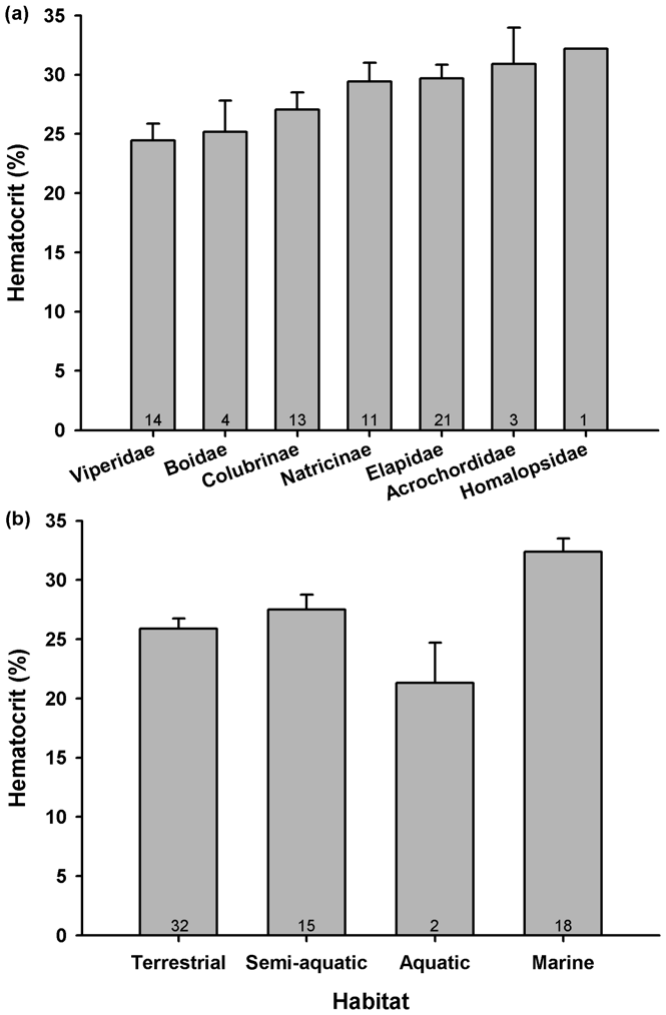
Hct (simple mean ± SE) in relation to family (a) and habitat (b). Numbers in the bars indicate sample size (number of species).

Based on partial F tests, both the OLS and RegOU models indicate that with effects of Habitat controlled, Acrochordidae have significantly elevated levels of Hct relative to the Colubrinae clade (Table 2, Figure 2a). Additionally, the Natricinae clade (entirely semi-aquatic) tends to have higher Hct than Colubrinae (entirely terrestrial; marginally not significant for OLS, and significant for RegOU, Table 2, Figure 2a). With effects of Clade controlled, aquatic snakes had significantly lower Hct than their terrestrial counterparts, whereas marine snakes display significantly higher Hct (Table 2, Figure 2b).

**Table 2:** Full models to predict Hct.

	Conventional (OLS)					Phylogenetic with OU transform				
Variable	Coefficient	SE	F	df	P	Coefficient	SE	F	df	P
Y-intercept	27.0462	1.120				26.7951	1.259			
Semi-aquatic	**−**3.3531	2.381	1.98	1, 57	0.1648	**−**3.8318	2.395	2.559	1, 57	0.1152
Aquatic	**−**23.4794	5.447	18.58	1, 57	<0.0001	**−**23.5633	5.571	17.891	1, 57	<0.0001
Marine	5.3206	2.278	5.45	1, 57	0.0231	5.2367	2.363	4.911	1, 57	0.0307
Boidae	**−**1.0329	2.385	0.19	1, 57	0.6646	**−**0.9355	2.600	0.129	1, 57	0.7208
Acrochordidae	17.7332	4.771	13.81	1, 57	0.0005	18.068	4.897	13.612	1, 57	0.0005
Viperidae	**−**2.3495	1.565	2.25	1, 57	0.1391	**−**2.056	1.750	1.381	1, 57	0.2448
Homalopsidae	**−**0.1668	4.771	<0.01	1, 57	0.9723	**−**0.1682	4.897	<0.01	1, 57	0.9728
Elapidae	**−**1.0949	2.329	0.22	1, 57	0.6408	**−**0.7096	2.434	0.085	1, 57	0.7717
Natricinae	5.7192	2.900	3.89	1, 57	0.0534	6.2637	3.019	4.304	1, 57	0.0425
Habitat			15.41	3, 57	<0.0001			14.69	3, 57	<0.0001
Clade			5.30	6, 57	0.0002			4.92	6, 57	0.0004

Although OLS is the preferred model based on AICc (Table 1), we also show the next-best RegOU model. Note that the base groups for Habitat and Clade are Terrestrial and Colubrinae, respectively. Partial regression coefficients and significance levels are relative to those base groups. See text and [21], [27] for further explanations.

## Discussion

Both Clade and especially Habitat accounted for a substantial amount of the variation in Hct (Tables 1, 2). A significant effect of Clade on a non-hierarchical tree (OLS model) indicates that hematocrit varies among major branches of the tree, while the fact that the OLS model fits better than either PGLS or RegOU models indicates no statistically significant "phylogenetic signal" [20], [21], [27], [29] remains in the residuals after accounting for Clade and Habitat.

Contrary to previous findings [9], [12], [13], [31], our study suggests that marine snakes display increased blood-oxygen stores. For instance, marine elapids, the most important and diverse sea snake lineage (N = 16 species, mean Hct 31.3%) had higher Hct than terrestrial elapids (N = 3 species, mean Hct 25.6%). Due to constraints of marine environments (versus shallower, freshwater environments), marine snakes rely on potentially deep (>80 m) and long (>1 h) active dives [4], [32]. In this respect, increased Hct might allow prolonged dive duration and sustained aerobic metabolism even during the longest dives [14]. In turn, long dives would allow marine snakes to remain in contact with potential prey for greater periods of time (or to prolong courtship duration [10]), but also to surface less frequently to replenish oxygen stores, thereby decreasing predation risks while these conspicuous species travel through the water column. Acrochordids also showed significantly elevated Hct, probably because the marine *Acrochordus granulatus* has the highest Hct in our dataset (>50% *versus* ∼21% for the two other Acrochordidae in our dataset; see below). Blood-oxygen carrying capacities of *Acrochordus granulatus* exceed those of other reptiles and approach levels characteristic of endothermic mammals and birds [16]. Determining whether this peculiarity is related to the marine habits of *A. granulatus* versus other ecological specificities (e.g., use of calm, anoxic water bodies [16]) will require further investigations (see below).

Although our results also suggest that aquatic species have lower Hct than their terrestrial counterparts (Table 2), we recommend these results be interpreted with caution. The aquatic category contained our lowest sample size (N = 2) and both species belong to the Acrochordidae (*Acrochordus arafurae* 21.6%, *Acrochordus javanicus* 21.0%), a lineage known to be very atypical in terms of ecology, morphology, and physiology compared to other Caenophidia [30]. Future studies should examine Hct of other fully aquatic snake species. In keeping with this cautious interpretation, further examination of our data set indicates that semi-aquatic colubrids (Natricinae) tended to have slightly higher Hct than their terrestrial counterparts (29.4% vs 27.1%).

Our current analysis does not allow us to discern the respective contributions of diving habits *per se* and other oxygen-demanding habits on increased Hct in marine snakes (notably due to a lack of data on diving freshwater species; see above). For instance, marine reptiles display specific physiological adaptations related to osmoregulation (i.e. salt-glands [33]). Expelling excess salt in a hyper-osmotic medium such as seawater is costly in term of energy, and thus oxygen requirements ([34] but see [15]). A superficial examination of the data from Acrochordidae could lead to a picture where the freshwater *A. arafurae* and the facultatively brackish-water *A. javanicus* both display lower Hct than the closely related marine *A. granulatus* (i.e. ∼21% *versus* >50%). However, this example does not allow robust conclusions about marine snakes in general, most notably because of the atypical ecology, morphology, and physiology of this lineage. Future exploration should focus on a promising “model lineage” of highly aquatic snakes - Homalopsidae, especially the widespread *Cerberus rynchops* - in which different populations of the same species use a continuum of contrasting aquatic habitats (freshwater, brackish, and saltwater [35]; see also [36], [37] for comparison between closely related Natricinae using freshwater versus estuarine habitats). Accordingly, other ecological aspects of snake life history might also benefit from increased Hct and thus increased aerobic capacity. For instance, contrasting activity levels (e.g. active versus sit-and-wait foraging) might have a strong impact on the evolution of snake locomotor performance, Hct, and other cardiovascular and respiratory characteristics. We suggest that future studies should explore snake eco-physiology in relation to activity levels, independently from habitat use [38].

Alternatively, increased Hct is also likely to impose costs, especially in terms of increased blood viscosity. Such issues have been investigated in the case of *A. granulatus*, for which higher blood viscosity is at least partially compensated by unusually low metabolic rates and sluggish habits [9], [16], [30]. However, we suggest that the great overlap of Hct among habitats (e.g. Figure 1) does not suggest a specific effect of blood viscosity (and its potential effects on aspects of the cardiovascular system) for most marine snakes (except *A. granulates*; see above). Overall, exploring the effects of increased blood viscosity on cardiovascular and respiratory systems (e.g. blood pressure, cardiac rhythms, metabolic rates) in snakes remains a largely open field of investigation.

Additionally, we acknowledge that our analysis does not take into account the role of the right lung (the left one being vestigial and non-functioning or totally absent in Caenophidia), which is obviously a major component of respiratory physiology [9]. Indeed, the lungs of marine elapids and acrochordids are longer than those of terrestrial snakes, extending from the neck to the posterior end of the body cavity [9]. Our limited knowledge of snake diving behaviour, however, does not indicate whether this elongated lung is used as an oxygen store while diving. Most notably, the inflation state of the lung during dives has not been quantified. Although an inflated lung would provide increased oxygen stores, it would also increase buoyancy and considerably increase the effort spent by a swimming snake to reach the seafloor to forage. Clearly, investigations on the role of the lung as an organ used for buoyancy regulation versus oxygen stores in marine snakes are needed.

Lastly, we note some limitations of our analysis. The use of Hct as a proxy for blood oxygen stores is a simplification, and other blood-related traits are likely to play a role in oxygen storage and distribution, and thus in the overall respiratory physiology of marine snakes. Most notably, blood volume is a likely candidate to examine in order to refine estimates of oxygen stores in snakes [9], [10], [12]–[14]. Although the few available values for blood volumes suggest a similarity between land and sea snakes ([12], [14] but see [16]), we emphasize that a comprehensive analysis of snake oxygen stores and habitat use will require a thorough investigation. Additionally, examinations of other traits, such as hemoglobin-oxygen affinity, or the role of cardiac shunts and cutaneous gas exchanges (all of which have been assessed for only a limited subset of species [9], [10], [12]–[14], [16], [39]), will be crucial in unravelling the respiratory challenges faced by snakes during the invasion of aquatic and marine ecosystems. This lack of information clearly points out the need for further investigations of the ecological and evolutionary physiology of marine and aquatic snakes.

Although our results show that marine snakes have increased Hct as compared with terrestrial species, this difference seems modest in comparison to what is found in marine endotherms [2], [3], despite the fact that marine snakes are extremely good divers [4], [18], [32]. Indeed, Figure 1 highlights the great overlap between Hct recorded in terrestrial and marine snakes. We emphasise that the relatively modest effects we document is a major phenomenon to explore. Although the generally low metabolic rates and great physiological flexibility of ectotherms are likely to explain such differences [4], [5], [15], future investigations are required to determine what other components of the cardiovascular and respiratory systems have been altered adaptively during the evolution of marine snakes [40], and whether such alterations may have led to trade-offs with other traits (e.g. the effects of blood viscosity; see above), including components of the life history [41].

## Supporting Information

Appendix S1 Online_Appendix_S1.xls. Data file in Excel file format with species code (PDICode) in column 1. Column 2 is the left to right order of taxa in our phylogenetic tree (Figure 1, see Online Appendix S2 and S3). Clade and Habitat are the classifications used in our analysis. Hct is percentage of hermatocrit per unit volume blood and Source indicates the source of our data (see Excel sheet Source for complete references).
(XLS)

Appendix S2 Details on the phylogeny construction.
(DOC)

Appendix S3 File of Phylogenetic tree. This file (Hct67P_2.phy) of the phylogenetic tree (described in Online Appendix S2, and shown in Figure 1) was produced in Mesquite (Version 2.72 Maddison and Maddison, 2006; http://mesquiteproject.org). It is in the Newick Standard File Format.
(PHY)
