# Supplementary material for: Is Aquatic Life Correlated with an Increased Hematocrit in Snakes?
Source: PLoS One. 2011 Feb 16;6(2):e17077. doi: 10.1371/journal.pone.0017077 (PMC3040194; doi:10.1371/journal.pone.0017077)
Supplement: Appendix S2 — Details on the phylogeny construction. (DOC) [file pone.0017077.s002.doc]

**Online Appendix S2.** Details on the phylogeny construction.

Most published trees contained only a few taxa of interest for any particular group. Therefore, we were often forced to use multiple trees which may have employed different characters and methods in their analyses to place particular taxa into our tree (e.g. Elapideae, see below).

Maximum likelihood and Bayesian trees were preferred over those obtained by other methods (e.g. parsimony), and strict consensus trees were used when available. Where several phylogenetic hypotheses exist for a given taxonomic group (particularly troublesome with species level relationships), we used the number of characters and the number of taxa to differentiate among them. For example, if two trees had similar numbers of characters, then we chose the one with more taxa (and vice-versa).

In a few instances, the amount of clade support (e.g., Bayesian posterior probabilities, bootstrap values, etc.) influenced our decision on tree selection and construction. We collapsed clades to soft polytomies to maintain a more conservative approach in those instances where no phylogenetic hypotheses could be found or where nodal support was particularly weak. For the genus *Laticauda*, we relied exclusively on morphological studies to construct species level relationships (Heatwole et al. 2005, Kharin & Czeblukov 2006).

The phylogenetic tree used for all analyses was constructed from previously published phylogenetic hypotheses of both high and low level relationships among snakes. The tree was constructed and manipulated in Mesquite (Maddison and Maddison 2009). All 65 species of snakes listed in the Online Appendix 1 are represented in the tree (Online Appendix 3), and for statistical analyses branch lengths were set by the arbitrary method of Pagel (1992).

For our purposes, we have used Vidal and Hedges (2004), Vidal et al. (2007a) and Wiens et al. (2008) as the basis for our higher-level relationships. These studies are in congruence with most other molecular studies and those that include morphological characters (e.g., Lee et al. 2007) in their placement of scolecophidians as sister to all other snakes and the monophyly of the Alethinophidia, Caenophidia, and Colubroidea.

We used the hypothesis of Vidal et al. (2007b) to resolve relationships among the Caenophidia. This tree was chosen over several others for both its strong nodal support and for the large number of genes used (seven nuclear protein coding genes versus two mitochondrial genes in Heise et al. 1995, 1 mitochondrial and 3 nuclear genes in Vidal and Hedges 2002, two nuclear genes in Vidal and Hedges 2004, and one nuclear and one mitochondrial gene in Lawson et al., 2005). In addition, nuclear protein-coding genes have proven to yield more consistent results in previous studies of higher level squamate phylogeny (Townsend et al. 2004; Vidal and Hedges 2004; Vidal and Hedges 2005). For our purposes, Caenophidia is defined as the Colubroidea (*sensu* Lawson et al. 2005) and its sister group, the Acrochordidae.

The following list shows within-family relationships and is constructed such that indentations are indicative of relative position in the phylogenetic hierarchy. For instance subfamilies such as Colubrinae or Natricinae would be indented relative to the Colubroidea because both of these subfamilies are subgroups of the more inclusive Colubroidea. Taxa that are indented to the same degree are not necessarily taxonomically equivalent (i.e., families, subfamilies and tribes may be indented to the same degree under a particular heading) nor does the particular ordering under a particular heading indicate any form of a nested relationship. The monophyly and sister relationships of groups of two or less taxa (e.g., *Acrochordus* spp., *Nerodia* spp.) was assumed.

*Boidae.* The relationships are from Burbrink (2005). The Pythons and Boas were placed sister to one another as they were the only two representative of the Henophidia.

*Colubrinae*. The ten species of Colubrid snakes in our tree form a monophyletic clade sister to two species of Xenodontines (*Heterodon contortrix* and *Heterodon platyrhinos*). The overall relationships of the major groups of the family come from Utiger et al. (2002) and Nagy et al. (2004). The relationship among the racers comes from Creer (2001) while the relationship among the Lampropeltini is from Pyron and Burbrink (2009). The relative position of *Boiga* is from Lawson et al. (2004).

*Natricinae*. The relationships among the Natricines are from Alfaro and Arnold (2001) and Guicking et al. (2006). The European *Natrix* form a clade sister to North American watersnakes (*Nerodia* and *Thamnophis*). The position of *Xenocrhophis* is sister to that of the North American Natricines.

*Elapidae.* The Elapids form two distinct clades, an Afro-Asian clade (1 species) and an Australo-Papuan clade (20 species). The latter clade is comprised of a terrestrial Australian Hydrophiinae group sister to the “true” seasnakes. This group forms the sister clade to the Laticaudid sea kraits. The relationships among the Elapids comes from Keogh (1998), Keogh et al. (2000), Lukoscheck & Keogh (2006), Sanders et al. (2008), Heatwole et al. (2005), Kharin & Czeblukov (2006).

*Viperidae*. The Vipers form two distinct well supported monophyletic clades (*Viperinae* and *Crotalinae*). Asian pit-vipers (one species) form the sister group to all new-world Crotalids. The major relationships between New World pit vipers comes from Castoe and Parkinson (2006) while relationships among the neo-tropical members of the group (*Bothrops*) comes from Wüster et al. (2002). The relationship among the rattlesnakes (*Crotalus*) comes from Murphy et al. (2002). The relationship among the true vipers (Viperinae *sensu stricto*) comes from Wüster et al. (2008).

**Literature cited in Appendix S2**

Alfaro, M. E. & Arnold, S. J. 2001 Molecular systematics and evolution of *Regina* and the Thamnophiine snakes. *Mol. Phyl. Evol.* **21**, 408-423.

Burbrink F.T. 2005. Inferring the phylogenetic position of Boa constrictor among the Boinae. Mol Phylogenet Evol 34:167–180.

Castoe, T. A. & Parkinson, C. L. 2006 Bayesian mixed models and the phylogeny of pitvipers (Viperidae: Serpentes). *Mol. Phyl. Evol.* **39**, 91–110

Guicking, D., Lawson, R., Joger, U. & Win, M. 2006 Evolution and phylogeny of the genus *Natrix* (Serpentes: Colubridae). *Biol. J. Linn. Soc.* **87**, 127-143

Heatwole, H., Busack, S. & Cogger, H. 2005 Geographic variation in sea kraits of the *Laticauda colubrina* complex (Serpentes: Elapidae: Hydrophiinae: Laticaudini). *Herpetol. Monogr.* **19**, 1-136

Kharin, V. E. & Czeblukov, V. P. 2006 A new revision of sea kraits of family Laticaudidae Cope, 1879 (serpentes: colubroidea). *Russ. J. Herpetol.* **13**, 227-241

Keogh, J. S. 1998 Molecular phylogeny of elapid snakes and a consideration of their biogeographic history. *Biol. J. Linn. Soc.* **63**, 177–203

Keogh, J. S., Scott, I. A. W. & Scanlon, J. D. 2000. Molecular phylogeny of viviparous Australian elapid snakes: affinities of *Echiopsis atriceps* (Storr, 1980) and *Drysdalia coronata* (Schlegel, 1837), with description of a new genus. *J. Zool.* **252**, 317-326

Lawson, R., Slowinski, J. B. & Burbrink, F. T. 2004 A molecular approach to discerning the phylogenetic placement of the enigmatic snake *Xenophidion schaeferi* among the Altethinophidia. *J. Zool.* **263**, 285-294.

Lawson, L. R., Slowinski, J. B., Crother, B. I., Burbrink, F. T. 2005 Phylogeny of the Colubroidea (Serpentes): New evidence from mitochondrial and nuclear genes. *Mol. Phyl. Evol.* **37**, 581–601

Lukoscheck, V. & Keogh, J. S. 2006. Molecular phylogeny of sea snakes reveals a rapidly diverged adaptive radiation. *Biol. J. Linn. Soc.* **89**, 523–539

Murphy, R. W., Fu, J., Lathrop, A., Feltham, J. V. & Kovac V. 2002 *Phylogeny of the rattlesnakes (Crotalus and Sistrurus) inferred from sequences of five mitochondrial DNA genes*. In Biology of Vipers (eds. G. W. Schuett, M. Höggren, M. E. Douglas & H. W. Greene), pp. 69-92. Eagle Mountain Publishing LC: Utah.

Nagy, Z. T., Lawson, R., Joger, U. & Wink, M. 2004 Molecular systematics of racers, whipsnakes and relatives (Reptilia: Colubridae) using mitochondrial and nuclear markers. *J. Zool. Syst. Evol. Res.* **42**, 223–233

Pagel, M. D. 1992 A method for the analysis of comparative data. *J. Theor. Biol.* **21**, 431-442.

Pyron, R. A. & Burbrink F. T. 2009 Neogene diversification and taxonomic stability in the snake tribe Lampropeltini (Serpentes: Colubridae). *Mol. Phyl. Evol.* **52**, 524–529

Sanders, K. L., Lee, M. S. Y., Leys, R., Foster, R. & Keogh, J. S. 2008 Molecular phylogeny and divergence dates for Australasian elapids and sea snakes (Hydrophiinae): evidence from seven genes for rapid evolutionary radiations. *J. Evol. Biol.* **21**, 682-695

Utiger, U., Helfenberger, N., Schätti, B., Scmidt, C., Ruf, M. & Ziswiler, V. 2002 Molecular systematics and phylogeny of old and new world ratsnakes, *Elaphe* Auct., and related genera (Reptilia, Squamata, Colubridae). *Russ J Herpetol.* **9**, 105-124

Vidal, N. & Hedges, S. B. 2002 Higher-level relationships of snakes inferred from four nuclear and mitochondrial genes. *C. R. Biologies* **325**, 977-985.

Vidal, N. & Hedges, S. B. 2004 Molecular evidence for a terrestrial origin of snakes. *Biol. Lett.* **271**, S226–S229

Vidal, N. & Hedges, S. B. 2005 The phylogeny of squamate reptiles (lizards, snakes, and amphisbaenians) inferred from nine nuclear protein-coding genes. *C. R. Biologies* **328**, 1000–1008

Vidal, N., Delmas, A.-S. & Hedges, B. S. 2007a *The higher-level relationships of alethinophidian snakes inferred from seven nuclear and mitochondrial genes*. In Biology of the Boas and Pythons (eds R. W. Henderson & R. Powell), pp. 27-33 Eagle Mountain Publishing LC: Utah.

Vidal, N., Delmas, A.-S., David, P., Cruaud, C., Couloux, A. & Hedges, S. B. 2007b The phylogeny and classification of caenophidian snakes inferred from seven nuclear protein-coding genes. *C. R. Biologies* **330**, 182-187.

Wiens, J. J., Kuczynksi, C. A., Smith, S. A., Mulcahy, D. G., Sites, J. W. Jr., Townsend, T. M. & Reeder, T. W. 2008 Branch lengths, support, and congruence: Testing the phylogenomic approach with 20 nuclear loci in snakes. *Syst. Biol.* **57**, 420-431

Wüster, W., Salomão, M. D. G., Quijada-Mascareñas, J. A., Thorpe, R. S. & BBBSP. 2002 *Origins and evolution of the South American pitviper fauna: Evidence from mitochondrial DNA sequence analysis*. In Biology of Vipers (eds. G. W. Schuett, M. Höggren, M. E. Douglas & H. W. Greene), pp. 111-128. Eagle Mountain Publishing LC: Utah.

Wüster, W., Peppin, L., Pook, C. E. & Walker, D. E. 2008 A nesting of vipers: Phylogeny and historical biogeography of the Viperidae (Squamata: Serpentes). *Mol. Phylo. Evol.* **49**, 445-459.
